# Characterization of Lgr5+ progenitor cell transcriptomes in the apical and basal turns of the mouse cochlea

**DOI:** 10.18632/oncotarget.8636

**Published:** 2016-04-07

**Authors:** Muhammad Waqas, Luo Guo, Shasha Zhang, Yan Chen, Xiaoli Zhang, Lei Wang, Mingliang Tang, Haibo Shi, Phillip I. Bird, Huawei Li, Renjie Chai

**Affiliations:** ^1^ State Key Laboratory of Bioelectronics, Institute of Life Sciences, Southeast University, Nanjing, China; ^2^ MOE Key Laboratory of Developmental Genes and Human Disease, Institute of Life Sciences, Southeast University, Nanjing, China; ^3^ Co-Innovation Center of Neuroregeneration, Nantong University, Nantong, China; ^4^ Department of Otorhinolaryngology, Hearing Research Institute, Affiliated Eye and ENT Hospital of Fudan University, Shanghai, China; ^5^ Central Laboratory, Affiliated Eye and ENT Hospital of Fudan University, Shanghai, China; ^6^ Institutes of Biomedical Sciences, Fudan University, Shanghai, China; ^7^ Department of Otolaryngology, The Affiliated Drum Tower Hospital of Nanjing University Medical School, Nanjing, China; ^8^ Department of Otolaryngology, Shanghai Jiao Tong University Affiliated Sixth People's Hospital, Otolaryngology Institute of Shanghai Jiao Tong University, Shanghai, China; ^9^ Department of Biochemistry and Molecular Biology, Monash University, Melbourne, Victoria, Australia

**Keywords:** Wnt signaling, Lgr5, cochlea, regeneration, proliferation

## Abstract

Lgr5+ supporting cells (SCs) are enriched hair cell (HC) progenitors in the cochlea, and several studies have shown a difference in the proliferation and HC regeneration ability of SCs between the apical and basal turns. However, the detailed differences between the transcriptomes of the apical and basal Lgr5+ SCs have not yet been investigated. We found that when isolated by FACS, Lgr5+ cells from the apex generated significantly more HCs and had significantly higher proliferation and mitotic HC regeneration ability compared to those from the base. Next, we used microarray analysis to determine the transcriptome expression profiles of Lgr5+ progenitors from the apex and the base. We first analyzed the genes that were enriched and differentially expressed in Lgr5+ progenitors from the apex and the base. Then we analyzed the cell cycle genes and the transcription factors that might regulate the proliferation and differentiation of Lgr5+ progenitors. Lastly, to further analyze the role of differentially expressed genes and to gain an overall view of the gene network in cochlear HC regeneration, we created a protein-protein interaction network. Our datasets suggest the possible genes that might regulate the proliferation and HC regeneration ability of Lgr5+ progenitors, and these genes might provide new therapeutic targets for HC regeneration in the future.

## INTRODUCTION

Sensorineural hearing loss is the leading cause of deafness in humans and has been a serious concern globally. In non-mammalian vertebrates, degeneration of HCs stimulates the surrounding SCs to acquire the HC phenotype, and this leads to spontaneous rebuilding of both the auditory and vestibular systems [1–3]. In mammals, the cochlear SCs in newborns contain HC progenitors and have a limited capacity to regenerate HCs through both direct differentiation and mitotic regeneration upon damage [4–8]. After maturation, however, the loss of HCs tends to be permanent due to a lack of regenerative ability [9–11].

In the mouse inner ear, SCs have been shown to be a reliable source for regenerating HCs after damage. Although the sensory epithelium is postmitotic, the SCs isolated from the postnatal cochlea possess the ability to proliferate and to subsequently differentiate into HCs *in vitro* [12, 13]. Upon damage, cochlear SCs also have a limited ability to proliferate, which leads to the mitotic regeneration of HCs [14, 15]. In addition, Notch inhibition [14, 16, 17], Wnt overexpression [7, 15, 18, 19], or Atoh1 overexpression [20–22] can induce SCs to generate more HCs via either direct differentiation or mitotic regeneration. Multiple studies have noted that the SCs in the apical turn have higher HC regeneration capacity than those in the basal turn [14, 15, 23], and we speculate that this might be because the apex is more immature than the base. However, the detailed gene expression profile differences between SCs in the apical and basal turns have not been investigated yet.

Lgr5 is a stable stem cell marker that is expressed in a subpopulation of cochlear SCs [24]. Lgr5+ cells have been shown to be an enriched population of progenitors in the cochlea that can regenerate HCs via both direct differentiation and mitotic regeneration [4, 6, 15, 25]. Our previous studies have noted that the Lgr5+ progenitor cells in the apex have higher HC regeneration ability than those in the base [6, 15], thus it is important to understand the detailed mechanism regulating these progenitor cells' proliferation and differentiation because these might provide new targets for inducing these progenitors to regenerate more HCs. However, there is no information available about the detailed differential gene expression or the fate of the Lgr5+ cells that are found in the apical and basal turns of the neonatal cochlea.

In the present study, we performed a detailed comparison between the Lgr5+ progenitors from the apex and the base. We found that Lgr5+ progenitors located in the apical turn of the neonatal cochlea displayed a significantly higher capacity to proliferate and regenerate HC than those in the basal turn. We further investigated the transcriptome expression profiles of Lgr5+ progenitors from the apex and the base to determine if any of the differentially expressed genes were involved in regulating proliferation, differentiation, or signaling pathways. Lastly we constructed a protein-protein interaction network using STRING (Search Tool for the Retrieval of Interacting Genes/Proteins) for analyzing the function of differentially expressed genes in inner ear HC regeneration. These datasets are expected to serve as a resource for determining the detailed regulatory mechanisms of cochlear progenitor cells.

## RESULTS

### Lgr5+ progenitors in the apex generate significantly more HCs *in vivo* compared with those in the base

**Figure 1:**
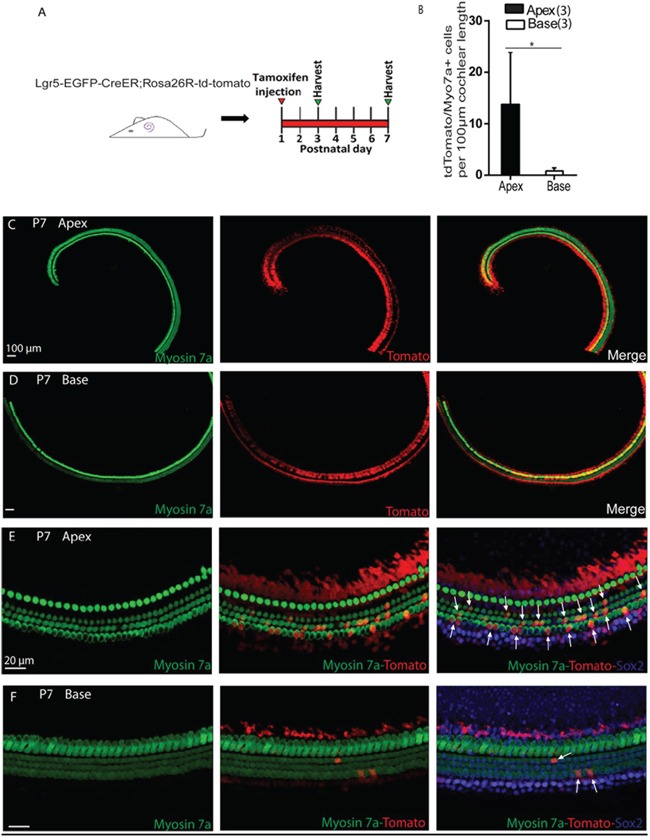
*In vivo* lineage tracing of Lgr5+ cells in the apical and basal turns of the postnatal cochlea **A.** Tamoxifen was injected intraperitoneally into P1 Lgr5-EGFP-creER/Rosa26-tdTomato mice, and the apical and basal regions were examined at P3 and P7. **B.** Counting data showed significantly higher numbers of tdTomato and tdTomato/Myo7a+ cells in the apex than in the base of the postnatal cochlea. **C** and **D.** Low-magnification images of the apical and basal regions show the expression of tdTomato and Myo7a. **E.** Traced tdTomato/Myo7a+ cells were found in the outer hair cell subset (arrow) in the apex. **F.** Few traced tdTomato/Myo7a+ cells were observed in the base. *p < 0.01. In panel B, *n* is shown in parentheses. Scale bars are 20 μm in C-F.

Cochlear Lgr5+ progenitors can generate HCs in the neonatal mouse *in vivo* [6, 25, 26]. First we identified the Lgr5-EGFP expression in the apical and the basal turn of the postnatal day (P)2 mouse cochlea. We observed Lgr5-EGFP expression in the third row of Deiters' cells, inner pillar cells, inner phalangeal cells, and the greater epithelium region (GER) in both the apex and the base. However, there are more Lgr5-EGFP+ cells in the GER in the apex than the base (Supplementary Figure 1A–1D). Next, we performed a lineage-tracing experiment by crossing Lgr5-EGFP-creER with the Rosa26-tdTomato reporter strain [27]. Tamoxifen was administered at P1, and cochleae were harvested and examined at P3 and P7 (Figure 1A). Consistent with previous reports, expression of the tdTomato reporter was first observed in Lgr5+ SCs in both the apical and basal turns at P3 [6]. When the period of tracing was prolonged to P7, significantly more tdTomato/Myo7a double-positive cells were observed in the apical turn than the basal turn (13.88 ± 3.27 and 0.83 ± 0.37 tdTomato/Myo7a double-positive cells per 100 μm length in the apex and base, respectively, *p* < 0.01, n = 3) (Figure 1B–1F), suggesting that the Lgr5+ progenitors in the apex generated significantly more HCs than those in the base *in vivo*.

### Lgr5+ progenitors in the apex generate more HCs compared to those in the base *in vitro*

**Figure 2:**
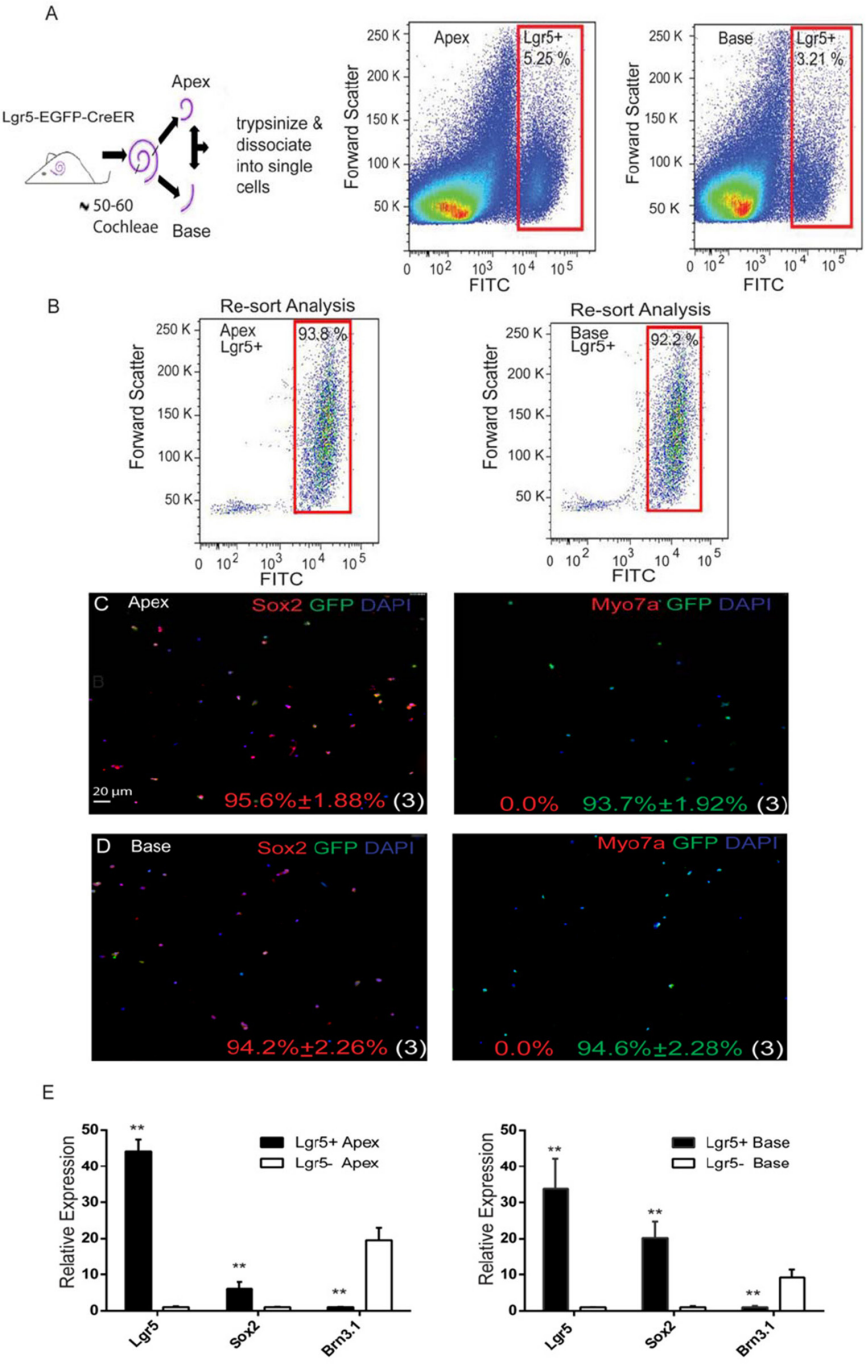
Re-sort analysis, immunostaining, and quantitative PCR of flow-sorted Lgr5+ cells from the apical and basal turns of the postnatal cochlea **A.** Lgr5-EGFP-CreER cochleae were dissected and separated into apical and basal fractions, and GFP+ and GFP− cells from each fraction were sorted by flow cytometry. **B.** Re-sort analysis of ALPs and BLPs demonstrated >90% purity. **C.** Immediate immunostaining after sorting of Lgr5+ cells from the apex showed a high percentage of Sox2+ (95.6%) and GFP+ (93%) cells but no Myo7a+ cells (0.0%). **D.** Immunostaining of Lgr5+ cells from the base also showed a high percentage of Sox2+ (94.2%) and GFP+ (94.6%) cells, and no Myo7a+ (0.0%) cells were found in the sorted cells. **E.** Quantitative PCR results showed the relative expression of Lgr5, Sox2, and Brn3.1 in ALPs and BLPs.

**Figure 3:**
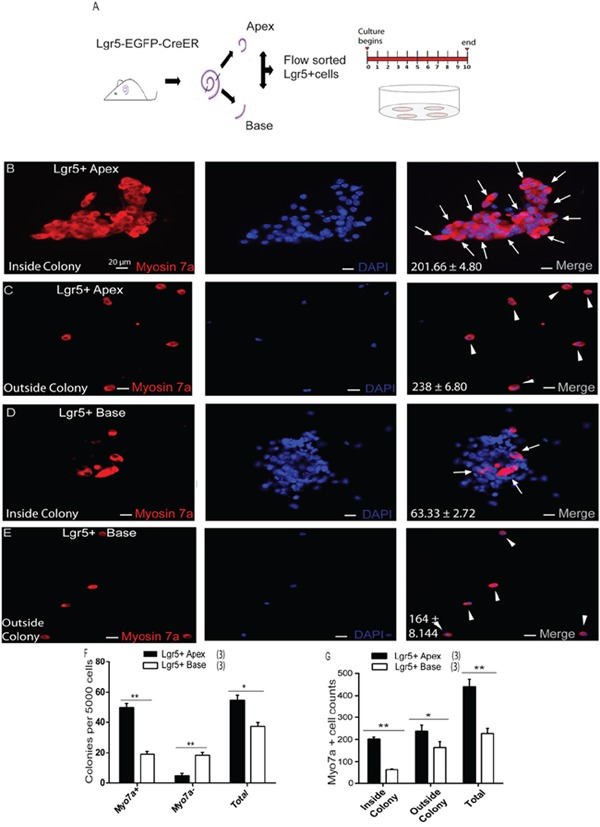
Lgr5+ SCs in the apex acted as hair cell progenitors *in vitro* **A.** Lgr5+ cells from the apical and basal turns of the cochlea were sorted by flow cytometry. **B.** Lgr5+ cells were isolated from the apex of Lgr5-EGFP-CreER mice culture for 10 days, and the majority of cells generated Myo7a+ hair cells (arrows) inside the colony. DAPI was used to stain the nuclei. **C.** More Myo7a+ hair cells (arrowheads) were also observed outside the colony. **D.** Lgr5+ cells were isolated from the base of the cochlea of Lgr5-EGFP-CreER mice cultured for 10 days, and only a few cells generated Myo7a+ hair cells (arrows) inside the colony. **E.** Relatively fewer Myo7a+ hair cells (arrowhead) were observed outside the colony. **F.** Lgr5+ cells from the apex formed more colonies than those from the base. Ninety percent of the colonies from the apex contained Myo7a+ cells. **G.** Lgr5+ cells from the apex generated significantly more Myo7a+ cells inside the colony compared with the base. Data are presented as mean ± SD. *p < 0.01; **p < 0.001. In panels F and G, *n* is shown in parentheses. Scale bars are 20 μm in B-E.

In order to investigate the HC regeneration ability of apical Lgr5+ progenitors (ALPs) and basal Lgr5+ progenitors (BLPs), we genotyped P1–P2 Lgr5-EGFP-Cre-ER mice, isolated the cochleae, and split the cochleae into equal fractions of apical and basal turns before dissociation of the cells. We sorted out the GFP+ cells from each fraction via flow cytometry, and these made up 5.25 ± 0.61% of the viable cells in the apical turn and 3.21 ± 0.35% of the viable cells in the basal fraction (Figure 2A). This was consistent with our immunohistochemistry data at P2, in which the GER in the apex contained more Lgr5-EGFP+ cells than the base (Supplementary Figure 1A–1D). Immunostained ALPs were 93.7 ± 1.92% GFP+, 95.6 ± 1.88% Sox2+, and 0% Myo7a+ (Figure 2C). Likewise, BLPs were 94.6 ± 2.28% GFP+, 94.2 ± 2.26% Sox2+, and 0% Myo7a+ (Figure 2D). Quantitative RT-PCR showed higher expression levels of Lgr5 and Sox2 and lower levels of the HC marker Brn3.1 in Lgr5+ cells compared to Lgr5− cells (Figure 2E). These data showed that the flow-sorted ALPs and BLPs were of high purity. To examine the HC regeneration capability of ALPs and BLPs, we cultured 5,000 cells in laminin-coated 4-well dishes at a density of 50 cells/μl for 10 days in serum-free medium and then immunostained them with the HC marker Myo7a. We found that the ALPs generated significantly more total colonies than the BLPs (5000 ALPs and BLPs generated 54.66 ± 2.02 and 37.33 ± 1.45 colonies, respectively, *p* < 0.01, n = 3) (Figure 3F); moreover, the number of Myo7a+ colonies was significantly higher in the ALPs, while the number of Myo7a− colonies was significantly higher in the BLPs (5000 ALPs and BLPs generated 48.33 ± 1.52 and 18.33 ± 1.20 Myo7a+ colonies and 4.66 ± 0.88 and 18.33 ± 1.20 Myo7a− colonies, respectively, *p* < 0.001, n = 3) (Figure 3F). We also found that ALPs could generate large epithelial colonies that expressed high levels of Myo7a (Figure 3B), which was never observed in the BLPs (Figure 2D). Furthermore, we characterized and counted the Myo7a+ cells inside and outside of the epithelial colonies. Compared with the BLPs, the ALPs regenerated significantly more HCs inside of the colonies, which represent the mitotically regenerated HCs (5000 ALPs and BLPs generated 201.66 ± 4.80 and 63.33 ± 2.72 HCs inside of the colonies, respectively, *p* < 0.001, n = 3) (Figure 3B, 3D, 3G). ALPs also regenerated significantly more HCs outside of the epithelial colonies, which represent the directly differentiated HCs (5000 ALPs and BLPs generated 238 ± 6.80 and 164 ± 8.144 HCs outside of the colonies, respectively, *p* < 0.01, n = 3) (Figure 3C, 3E, 3G). These results suggest that ALPs generate significantly more HCs compared with those from the BLPs *in vitro*, thus ALPs might serve as an enriched population of HC progenitors.

### Lgr5+ progenitors in the apex have a greater capacity to mitotically regenerate HCs compared with those in the base *in vitro*

**Figure 4:**
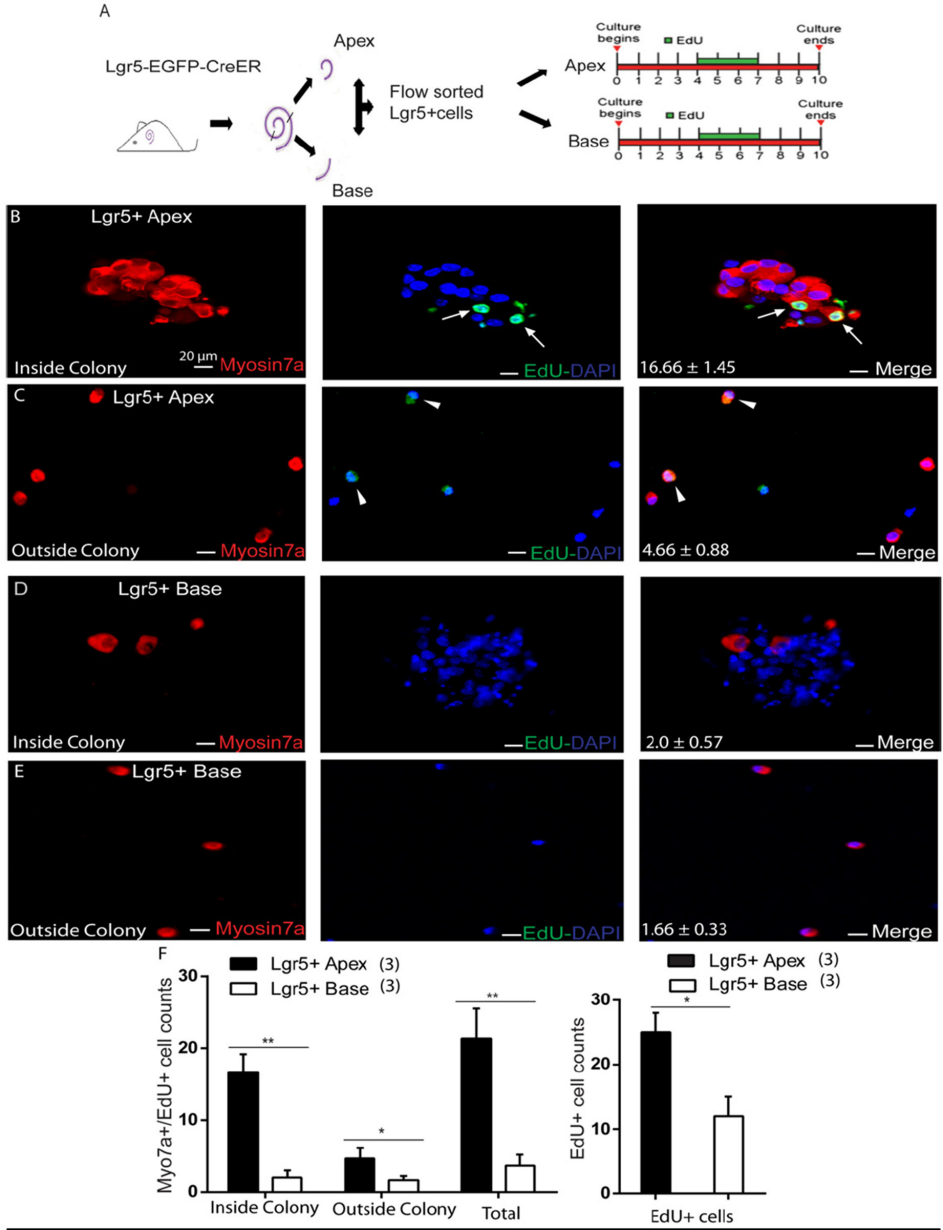
EdU labeling measures the proliferation ability of Lgr5+ cells from the apex and the base **A.** Flow-sorted Lgr5+ cells from cultures of the apex and base for 10 days (EdU was included in the culture from day 4 to day 7). **B.** Lgr5+ cells from the apex produced more Myo7a+/Edu+ cells (arrow) inside the colony. **C.** Fewer Myo7a+/Edu+ cells were observed outside the colony (arrowhead). **D** and **E.** Lgr5+ cells from the base showed a lack of Myo7a/EdU labeling inside and outside the colony **F.** Graph showing the significantly higher number of Myo7a+/EdU+ cells inside the colonies from the apex compared to the base, and only the EdU+ cell number was also comparatively higher in the apex than the base. In panel F, *n* is shown in parentheses. Data are presented as mean ± SD. *p < 0.01; **p < 0.001. Scale bars are 20 μm.

To determine the capacity of ALPs or BLPs to mitotically regenerate HCs, EdU was added to the culture medium from day 4 to day 7 during culture (Figure 4A). We counted the Myo7a+/EdU+ cells, which represent the mitotically regenerated HCs, and found that the majority of Myo7a+/EdU+ cells were inside the colonies and only a few of the Myo7a+/EdU+ cells were outside the colonies (Figure 4F). ALPs generated significantly more Myo7a+/EdU+ cells both inside and outside of the colonies compared to those from the BLPs (5000 ALPs and BLPs generated 16.66 ± 1.45 and 2.0 ± 0.57 Myo7a+/EdU+ cells inside of the colonies and 4.66 ± 0.88 and 1.66 ± 0.33 Myo7a+/EdU+ cells outside of the colonies, respectively, *p* < 0.01, n = 3) (Figure 4B–4F). Furthermore, we found that the total number of EdU+ cells was also noticeably higher in the apex than in the base (5000 ALPs and BLPs generated 25.0 ± 2.08 and 12.0 ± 1.52 EdU+ cells, respectively, *p* < 0.01, n = 3) (Figure 4F). These results indicate that ALPs have a greater capacity to mitotically regenerate HCs compared to BLPs.

### Lgr5+ progenitors from the apex have higher sphere-forming ability compared to those from the base *in vitro*

**Figure 5:**
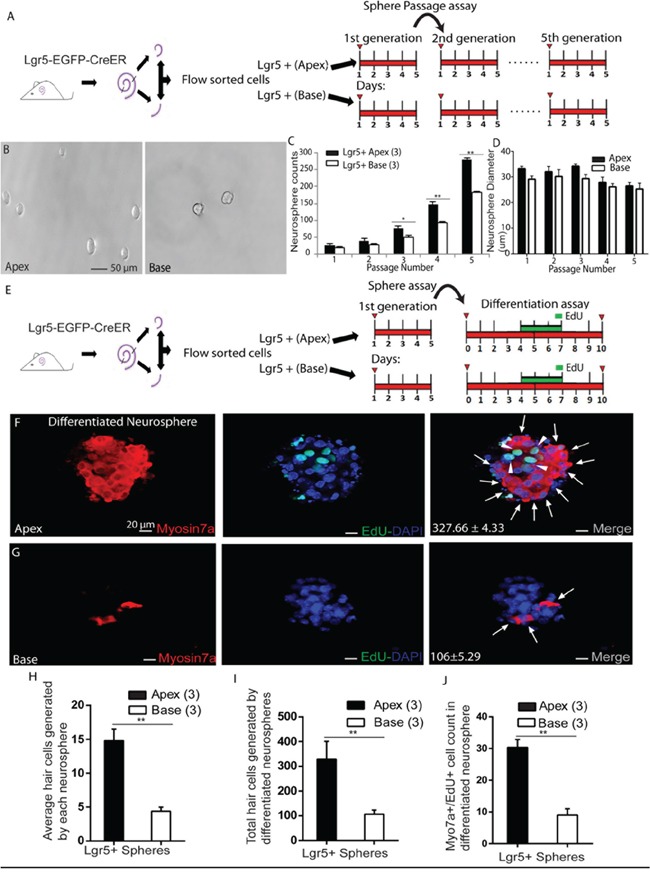
Neurosphere passage and differentiation assay **A.** Flow-sorted apical and basal Lgr5+ cells cultured for 5 days in ultra-low-attachment dishes for sphere passage assay. **B.** Lgr5+ cells from the apex generated significantly more neurospheres than those from the base. **C.** Neurospheres from Lgr5+ cells in the apex had a significantly higher rate of expansion than those from the base. **D.** No significant difference was observed in the diameter of neurospheres generated from Lgr5+ cells from the apex as compared to the base. **E.** Neurospheres derived from ALPs and BLPs were separated from the first generation to perform the differentiation assay. **F.** Upon differentiation of Lgr5+ neurospheres from the apex, a substantial proportion of Myo7a+ cells (arrows) were observed that also incorporated EdU (arrowhead). **G.** A smaller number of Myo7a+ cells (arrow) were observed upon differentiation of basal Lgr5+ neurospheres. **H.** Each Lgr5+ differentiated neurosphere from the apex generated significantly more Myo7a+ HCs than the base. **I.** Graph showing the significant difference in HC generation between apical and basal Lgr5+ neurospheres. **J.** More Myo7a+/EdU+ cells were found in differentiated neurospheres from the apex compared to the base. Data are presented as mean ± SD. In panels C, D, and G-I, *n* is shown in parentheses. *p < 0.05; **p < 0.01. Scale bars are 50 μm in B and 20 μm in E and F.

Sphere-forming ability has been used in multiple studies as one criterion to evaluate the cells' capacity as progenitors in the inner ear [6, 13, 23, 25, 26, 28]. Recent studies showed that Lgr5+ progenitors have higher sphere-forming ability and can generate more Myo7a+ HCs than other SCs [6, 25]. In order to specifically investigate the sphere-forming ability of ALPs and BLPs, we performed a neurosphere assay. ALPs and BLPs were isolated by flow cytometry, and a total of 200 isolated cells were plated into a 96-well ultra-low attachment plate at a density of 2 cells/μl for 5 days (Figure 5A). We measured the proliferation capacity by quantifying the number of spheres generated in each passage and found that the Lgr5+ neurospheres from the apex had a significantly higher rate of expansion than those from the base when passaging to multiple generations (Figure 5B–5C, *p* < 0.05, n = 3). Although fewer neurospheres were generated from progenitors in the base, the neurospheres were the same size as those generated from progenitors from the apex (Figure 5D). The higher sphere-forming ability of ALPs suggests that they possess greater proliferation ability and might have higher HC regeneration potential. In order to further evaluate the HC regeneration ability of these spheres, we isolated the neurospheres derived from ALPs and BLPs from the first generation and differentiated those spheres for 10 days. EdU was added from day 4 to day 7 during the culture (Figure 5E). We counted the Myo7a+ HCs in each differentiated sphere and the total Myo7a+ HCs that originated from the 200 isolated cells, and we found that the apical Lgr5+ neurospheres gave rise to significantly more Myo7a+ cells than the basal Lgr5+ neurospheres (Each neurosphere derived from the ALPs and BLPs generated 14.81 ± 0.98 and 4.4 ± 0.33 HCs, respectively, *p* < 0.01, n = 3, and neurospheres derived from 200 isolated ALPs and BLPs generated 327.66 ± 4.33 and 106 ± 5.29 HCs, respectively, *p* < 0.01, n = 3) (Figure 5F–5I). We also counted the Myo7a+/EdU+ HCs and found that ALPs generated significantly more Myo7a+/EdU+ HCs than BLPs (neurospheres derived from 200 isolated ALPs and BLPs generated 30.33 ± 1.45 and 9.0 ± 1.15 Myo7a+/EdU+ HCs, respectively, *p* < 0.01, n = 3) (Figure 5J). In sum, these results support the notion that ALPs have a greater capacity to form neurospheres and to generate HCs than BLPs.

### Analysis of microarray results

Microarray analysis was performed to determine the gene expression profiles of flow cytometry-isolated ALPs and BLPs. The whole-transcript arrays included probe sets to measure the expression of mRNA and non-coding RNA transcripts. The 41,345 transcriptional units had fluorescent intensity readings that varied from 2.61 to 11,869.65 for ALPs and from 2.74 to 11,903.02 for BLPs. Because the expression of every transcriptional unit was measured by signal intensity, a cutoff baseline intensity level for the background was chosen as 16.3 by averaging the signals of antigenomic background probes from all six arrays. Sample clustering analysis was performed on genes expressed in at least one group to assess reproducibility. ALP and BLP groups were well clustered, and no outliers were detected (Figure 9D). After excluding control sequences and signals below the baseline, 16,375 and 16,125 transcripts were examined in the ALP and BLP groups, respectively, and 15,525 transcripts were expressed in both cell populations (Figure 9E).

### Genes enriched in ALPs or BLPs

**Figure 6:**
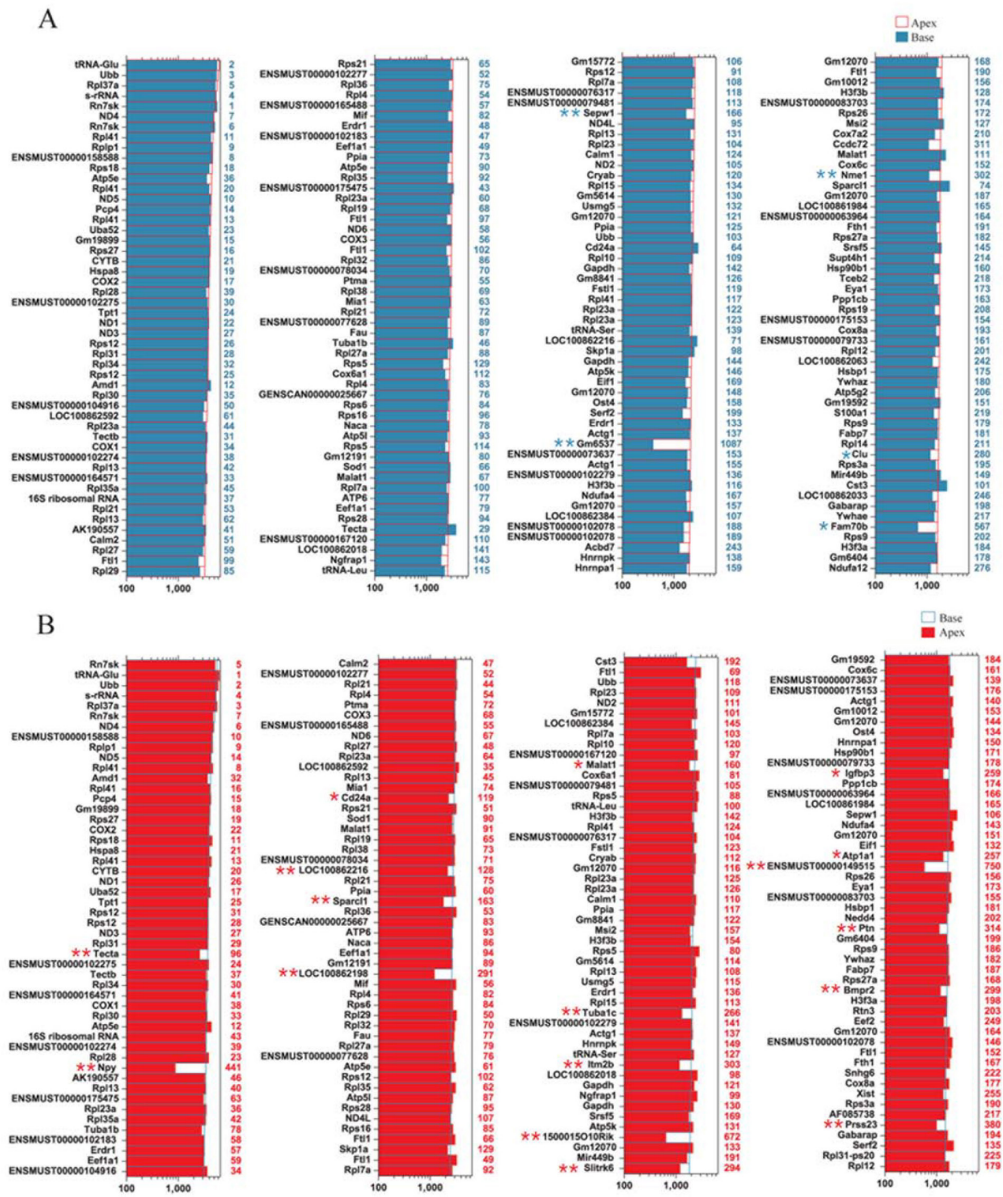
Expression levels of the top 200 genes in ALPs and BLPs **A.** Expression levels of the top 200 genes in ALPs in descending order. Numbers in blue on the right side of each panel represent the ranking of the same genes in BLPs. **B.** Expression levels of the top 200 genes in BLPs in descending order. Numbers in red on the right side of each panel represent the ranking of the same genes in ALPs.

In order to characterize the gene-expression profiles in ALPs and BLPs, we first analyzed the most abundantly expressed genes in both populations. Figure 6A shows the expression levels for the top 200 most abundant transcripts in the apical region. For comparison, expression levels for the same transcripts in the basal region and abundance rankings for these transcripts are also illustrated. Figure 6B similarly shows the 200 most abundant transcripts in BLPs compared to the same transcripts in ALPs. As shown in both figures, the majority of the transcripts that are richly expressed in one population are also abundantly expressed in the other. However, among the most abundantly expressed genes, Gm6537 and Fam70b were only richly expressed in ALPs, and Npy, Itm2b, 1500015O10Rik, ENSMUST00000149515, Ptn, and Prss23 were only richly expressed in BLPs.

### Differentially expressed genes in ALPs and BLPs

**Figure 7:**
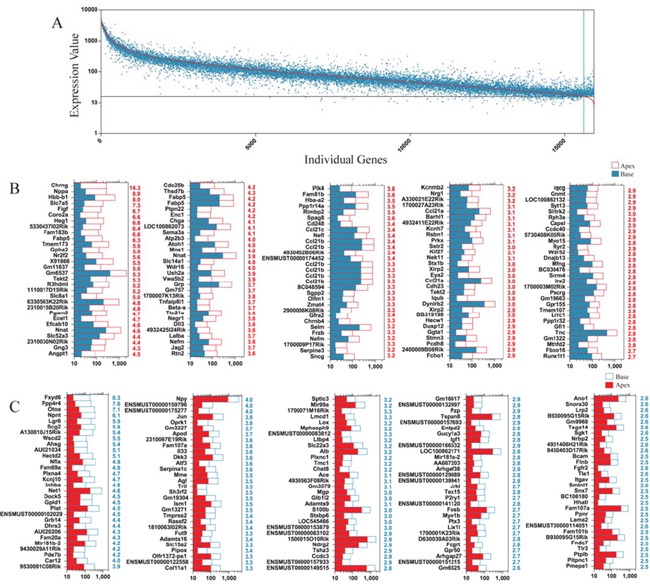
Differentially expressed genes in ALPs and BLPs **A.** All differentially expressed genes in ALPs and BLPs. The red line represents the expression level of 16,975 transcripts from ALPs, and each blue dot represents the expression level of the same transcripts from BLPs. **B.** The 150 most differentially expressed genes in 575 ALPs. The numerical values in red on the right side of each panel represent the fold difference in expression for ALPs versus BLPs. **C.** The 150 most differentially expressed genes in BLPs. The numerical values in red on the right side of each panel represent the fold difference in expression for BLPs versus ALPs.

To determine which genes are differentially expressed in ALPs and BLPs, we compared the expression levels of all the transcripts in ALPs with those of BLPs and selected the top differentially expressed genes in ALPs and BLPs. Figure 7A shows an overall picture of the expressed transcripts in ALPS and BLPs. Differentially expressed genes were categorized as those whose expression levels were above background and at least 1.5-fold different between the ALPs and BLPs (*p* < 0.05). We found 1,157 genes differentially highly expressed in ALPs, and 862 genes differentially highly expressed in BLPs. Figure 7B and 7C show the top 150 differentially expressed genes in ALPs and BLPs. The function of some of the differentially expressed genes has been reported previously. Some of the genes that are highly expressed in ALPs have been reported to play roles in inner ear HC development, differentiation, and patterning during embryonic and postnatal stages, including Atoh1, Dll3, Jag2, Barhl1, Gfi1, Nr2f2, Cdh23, Frzb, Xirp2, and Srrm4, which supports our notion that ALPs have a much greater potential to generate more sensory HCs in the neonatal cochlea and might partially explain why ALPs could generate more HCs than BLPs. However, a significant number of the differentially expressed genes have not been characterized before and need to be further studied in the future.

### Cell cycle analysis

**Figure 8:**
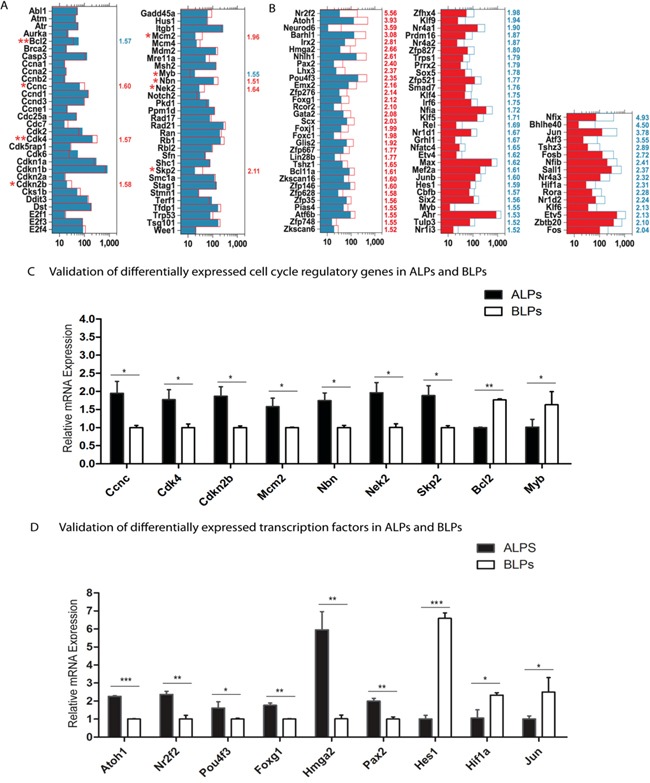
Genes regulating the cell cycle and transcription factors **A.** Expression levels of 60 genes that are important for cell cycle regulation. **B.** Expression levels of differentially expressed transcription factors. **C** and **D.** Quantitative RT-PCR analysis of the nine cell cycle regulatory genes and the nine transcription factors that are differentially highly expressed in ALPs and BLPs as identified by microarray analysis. Data are presented as the relative fold change in expression. Student's paired *t*-test; *p < 0.05; **p < 0.01; ***p < 0.001.

The mammalian cochlea has very limited capacity for spontaneous HC regeneration. In order to promote HC regeneration, it is important to induce HC progenitor cells to reenter the cell cycle and mitotically generate HCs. In the present study, we have demonstrated that ALPs have much greater ability to proliferate and mitotically generate HCs compared to BLPs; however, the detailed mechanism behind this difference remains unclear. To identify the possible genes regulating the cell cycling of Lgr5+ progenitors, we took advantage of microarray analysis to compare the expression of genes regulating the cell cycle and cell proliferation in ALPs and BLPs. It is reported that over 1,000 cell cycle genes might exist in the mammalian cell [29]. We examined the expression of 60 genes known to be involved in the cell cycle and that are commonly assayed in cell cycle PCR arrays. We found that Ccnc, Cdk4, Cdkn2b, Mcm2, Nbn, Nek2, and Skp2 were significantly highly expressed in ALPs and that Bcl2 and Myb were significantly highly expressed in BLPs (Figure 8A). To confirm the microarray results, we further performed qRT-PCR to validate the expression of these nine differentially expressed cell cycle genes. The qPCR data were consistent with the microarray analysis data, which also further validated the expression difference of these genes (Figure 8C). Skp2, one of the cell cycle-regulating genes that are highly expressed in ALPs, has been reported to play a role in stimulating cell proliferation during early inner ear development [30], and this gene might be involved in regulating the increased proliferation efficiency of ALPs compared to BLPs. However, most of the cell cycle-regulating genes that we identified in ALPs and BLPs have not been characterized before in the inner ear and need to be further studied.

### Transcription factors analysis

Transcription factors (TFs) are proteins that bind to either enhancer or promoter regions of genes thereby controlling the expression level of their target genes. TFs are involved in various processes throughout normal inner ear development. In the present study, we have demonstrated that ALPs have much higher HC regeneration capacity compared to BLPs; however, the precise roles of TFs in regulating the HC regeneration capacity of progenitors remain largely unknown. To determine which TFs might be involved in regulating HC differentiation from Lgr5+ progenitors, we examined the expression of 1,324 TFs in the mouse genome between the ALPs and BLPs. Figure 8B shows the 77 significantly differentially expressed TFs in ALPs and BLPs (*p* < 0.05, fold change > 1.5). Some of the TFs that are highly expressed in ALPs have been reported to play roles in promoting HC fate and patterning regulation during inner ear development, including Pou4f3, Atoh1, Nr2f2, Foxg1, Hmga2, and Pax2. Some of the TFs that are highly expressed in BLPs have been reported to be transcriptional repressors that inhibit the differentiation of HCs, including Hes1 [31], or have been reported to play critical roles in regulating cell survival and apoptosis in the inner ear, including Hif1 and Jun [32, 33]. Interestingly, we found that most of the reported TFs (as shown in Figure 8B) that promote HC regeneration are highly expressed in ALPs, which supports our hypothesis that these TFs might participate in the higher HC regeneration capacity of ALPs. Conversely, we observed higher expression of negative transcriptional regulators such as Hes1 and Jun in BLPs. To confirm the microarray results, we also performed qRT-PCR to validate the expression of these nine differentially expressed TFs, and the qPCR data is consistent with the microarray analysis data (Figure 8D). Furthermore, we have identified many TFs that have not been characterized before, and their involvement in the differential regeneration capacity of ALPs and BLPs should be investigated in the future.

### Gene ontology and network analysis of the genes differentially expressed in ALPs and BLPs

**Figure 9:**
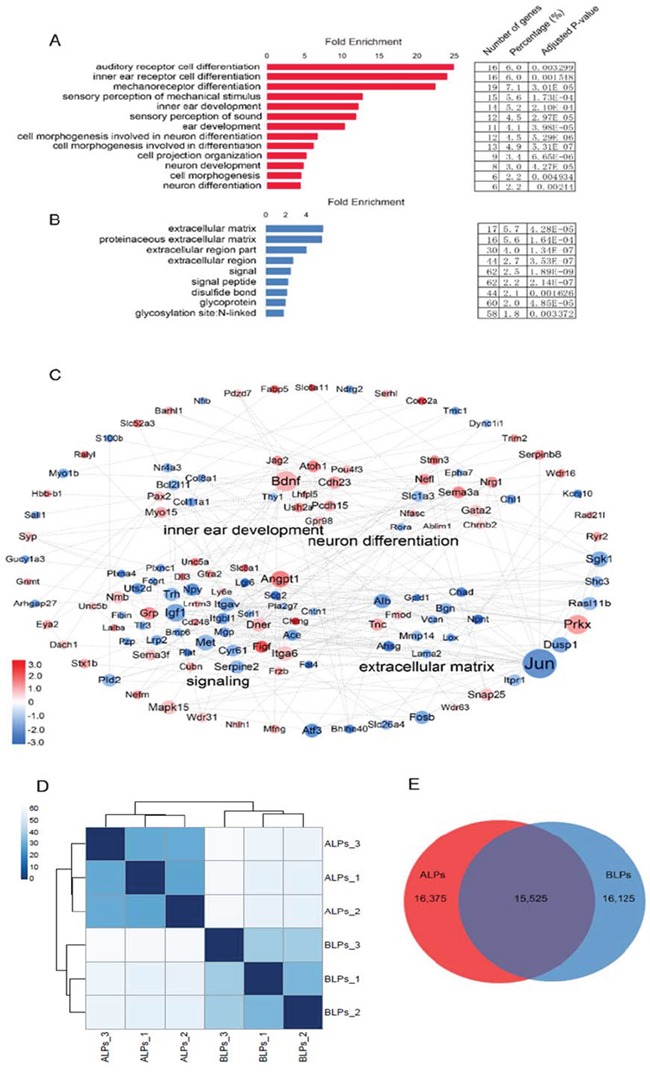
Gene ontology (GO) and network analysis of the genes differentially expressed in ALPs and BLPs, and PCA analysis **A.** GO analysis of genes differentially expressed in ALPs. **B.** GO analysis of genes differentially expressed in BLPs. **C.** STRING protein-protein interaction analysis of genes differentially expressed in ALPs (red) and BLPs (blue). The dashed lines indicate protein-protein interactions reported by the STRING analysis. The DAVID GO annotation was used to cluster the genes by biological function. **D.** Sample clustering analysis for all replicates of ALPs and BLPs. **E.** Venn diagram showing genes expressed in ALPs and BLPs.

To gain a comprehensive view of the gene network in inner ear HC regeneration, we combined a STRING protein-protein interaction analysis [34], which was used to assemble the predicted networks of the significantly altered genes (fold change > 2.0, *p* < 0.01) with the functional categories highlighted by gene ontology (GO) analysis (DAVID) (Figure 9C). This integrated analysis revealed a complex network of genes that are involved in inner ear HC development and are predicted to participate in regulating HC differentiation and function. GO analysis was also applied to genes up-regulated in ALPs or BLPs (fold change > 2.0, *p* < 0.01) (Figure 9A, 9B). As shown in Figure 9A, genes up-regulated in ALPs were highly enriched in functional categories such as HC differentiation and inner ear development, while the set of genes up-regulated in BLPs were slightly enriched in functional categories such as signaling and extracellular matrix.

## DISCUSSION

SCs have been demonstrated to be a reliable source for regenerating HCs. In the cochlea, Lgr5+ cells are the enriched population of HC progenitors compared to all SCs. Through lineage tracing, previous studies found that Lgr5+ progenitor cells in the apex generated more HCs than those in the base [6], and we speculated that this might be because cells in the apex are more immature compared to cells in the base. However, it is important to determine the detailed mechanisms regulating the proliferation and HC regeneration of Lgr5+ progenitors. Until now there have been no detailed comparisons between ALPs and BLPs, and the gene expression profiles in ALPs and BLPs have remained uninvestigated. Here, we found that the ALPs show much higher capacity to proliferate and regenerate HCs than BLPs. Furthermore, the neurospheres generated from ALPs showed a significantly higher rate of expansion and could differentiate to generate more HCs.

Recently, more and more attention has been focused on identifying the gene expression profiles in various mammalian cells. In the inner ear, several studies have reported the transcriptomes of HCs in mouse and zebrafish [35–38]. To further understand the mechanisms involved in the regulation of HC progenitors, it is necessary to identify the gene expression profiles of these cells. Here, we report the first genome-wide transcriptome analysis of purified ALPs and BLPs. First we analyzed the most abundantly expressed genes in ALPs and BLPs. We found that the majority of the top 200 richly expressed genes in ALPs and BLPs are similar, but two genes were significantly highly expressed in ALPs, and six genes were significantly highly expressed in BLPs. Among all the genes, only NPY, which is highly expressed in BLPs, was previously reported to participate in neuromodulation of afferent transmission in the inner ear [39].

### Differentially expressed genes in ALPs and BLPs

We demonstrated that ALPs had higher proliferation and HC regeneration capacity compared to BLPs. To further investigate the mechanisms behind this difference, we compared the differential gene expression between ALPs and BLPs. We found 2,019 genes that were expressed at significantly different levels between ALPs and BLPs. The majority of the differentially expressed genes have not been reported in the inner ear before, while some of them have been studied in previous reports. Genes highly expressed in ALPs include Atoh1, Tekt2, Dll3, Jag2, Barhl1, Gfi, Nr2f2, and TNC. Atoh1 alone is sufficient for HC development, and deletion of Atoh1 completely stops HC differentiation [40]. Barhl1 and Gfi1 are involved in HC differentiation during development [41, 42]. Nr2f2 has been implicated in cochlear patterning and HC differentiation [43]. The Cdh23, Frzb, and Xirp2 genes appear to promote an abrupt transition from progenitor cells to HCs [44–46]. Srrm4 has been reported as the first known alternative splicing regulator that is required for HC differentiation [47]. Our analysis showed that the expression of all of the aforementioned genes is crucial for cell proliferation and HC formation during inner ear development and that these genes might be the source of the proliferative and regenerative capabilities of ALPs. In addition, Dll3 and Jag2 are Notch ligands that prevent HC differentiation through lateral inhibition [48]. However, their expressions are also relatively higher in ALPs, and we speculate that this might be because the ALPs need higher expression of Notch signaling to prevent differentiation and thus maintain their progenitor capacity.

The set of previously reported genes that are highly expressed in BLPs includes Otoa, Lgr6, Igf1, Ano1, and Kcnj10. Otoa promotes the attachment of inner ear acellular gels, and Otoa mutations cause autosomal recessive deafness type 2 [49]. Lgr6 has been reported to have higher expression in the base, but the detailed function remains unclear [50]. Igf1 encodes an insulin-like growth factor that regulates the growth and development of the inner ear [51]. Ano1 encodes a transmembrane protein that functions as a calcium-activated chloride channel [52]. Kcnj10 is expressed in the stria vascularis and Deiters' cells and is crucial for cochlear development and maintaining extracellular potassium homeostasis [53]. Our findings suggest that the highly expressed genes in BLPs might be involved in the specific function of the basal cochlea but not in cell proliferation and HC regeneration.

### Cell cycle analysis

We showed that ALPs have higher capacity to proliferate and mitotically generate HCs than BLPs. To further characterize the genes regulating the proliferation of Lgr5+ progenitors, we examined 60 cell cycle regulation genes and found nine genes that were significantly differentially expressed between ALPs and BLPs. Among these genes, Skp2 has been found to be highly expressed in ALPs and to promote cell cycling. Up-regulation of Skp2 triggers the G1-S phase transition in precursor cells by regulating p27 and thus stimulates quiescent cochlear precursor cells to proliferate [30], and this suggests a potential role in cell cycle regulation in ALPs. In the present investigation, we also found other cell cycle-promoting genes, including Ccna2, Ccnd1, and cell cycle-inhibiting genes, including Cdkn1b, Cdkn1a, Rb1, that are abundantly expressed in both ALPs and BLPs. The other newly explored cell cycle regulatory genes identified in ALPs and BLPs still need to be further characterized.

### Transcription factor analysis

To further identify the TFs that regulate HC regeneration, we examined 1,324 TFs and found that 77 were significantly differentially expressed. Some of the reported TFs – including Pou4f3, Atoh1, Nr2f2, Foxg1, Hmga2, and Pax2 – are highly expressed in ALPs. Pou4f3 and Atoh1 have been reported to determine HC fate [40, 54]. The Nr2f2 gene is a downstream target of Pou4f3, and it is expressed most notably in the apical region [55]. Hmga2 is expressed along with Nr2f2 in the apical region [56]. Foxg1 promotes HC fate and patterning, and deletion of Foxg1 causes abnormal morphology and histology of the inner ear [57]. Similarly, Pax2 is a major patterning regulator during inner ear development, and its interaction with Eya1 decides the normal morphogenesis of the sensory area [58]. We found that the majority of the reported TFs that are highly expressed in ALPs are involved in HC development, differentiation, and patterning, and these might support the greater ability of ALPs to regenerate HCs.

Among the previously characterized TFs, Hes1, Hif1a, and Jun are highly expressed in BLPs. Hes1 is a transcriptional repressor that negatively regulates the differentiation of HCs by antagonizing Atoh1 expression [31], and this suggests that the higher expression of Hes1 in BLPs might suppress HC differentiation. Hif1 is a key TF required for cell survival under hypoxic conditions [32] and Jun plays a key role in apoptosis through the JNK pathway [33], indicating that the higher expression of Hif1 and Jun in BLPs might suppress the proliferation of BLPs. The other newly identified TFs in ALPs and BLPs still need to be further characterized.

### STRING prediction of inner ear HC development

We used the STRING database to construct a protein-protein interaction network for the differentially expressed genes between ALPs and BLPs, and the functional categories were highlighted by GO analysis. This integrated analysis revealed a complex network of genes that are predicted to participate in regulating HC differentiation. Importantly, ALPs and BLPs have significantly different expression levels of genes involved in inner ear development, neuron differentiation, and signaling pathways. In this network, most of the genes in the GO categories of inner ear development and neuron differentiation were highly expressed in ALPs, such as Atoh1, Pou4f3 and Cdh23, although several genes, e.g. Nr4a3 and Bcl2l11, were richly expressed in BLPs. Nr4a3 is a member of the nuclear receptor family, and suppression of Nr4a3 induces the migration and neurite extension of neuronal cells [59], and Bcl2l11 promotes apoptosis via the intrinsic mitochondrial pathway [60]. It would be interesting to investigate the involvement of these genes in regulating the progenitor cells in future.

In summary, we found that ALPs have greater capacity to proliferate and to regenerate HCs than BLPs. We next investigated the transcriptome differences between the ALPs and BLPs. We found several differentially expressed genes that might regulate the Lgr5+ progenitors' proliferation and HC regeneration capacity. Lastly, to further analyze the role of differentially expressed genes in HC regeneration, we constructed a STRING prediction map. The transcriptomes of ALPs and BLPs reported here establish a framework for future characterization of the genes that regulate the Lgr5+ progenitors and might provide new therapeutic targets for HC regeneration.

## MATERIALS AND METHODS

### Animals

We used Lgr5-EGFP-IRES-creERT2 mice (Stock #008875) [61] and Rosa26-tdTomato reporter mice of both sexes (Stock #007914) [27]. Both transgenic mice lines were acquired from The Jackson Laboratory. All animal procedures were performed according to protocols approved by the Animal Care and Use Committee of Southeast University and were consistent with the National Institutes of Health Guide for the Care and Use of Laboratory Animals. All efforts were made to minimize the number of animals used and to prevent their suffering.

### *In vivo* lineage tracing of Lgr5+ cells in the apical and basal turns of the cochlea

Lgr5-EGFP-creER mice (heterozygous) were crossed with Rosa26-tdTomato mice (homozygous) to trace the fate of Lgr5+ cells in the apical and basal turns of the cochlea. To activate cre, Lgr5-EGFP-creER/Rosa26-tdTomato double-positive mice were injected with tamoxifen (2 mg/25 g, Sigma) intraperitoneally at postnatal day (P)1. Mice were sacrificed, and the cochlear apex and base were examined at P3 and P7.

### Isolation of Lgr5+ cells via flow cytometry

Approximately 50–60 cochleae were dissected from Lgr5-EGFP-creER neonatal mice (P1–P2). The sensory epithelium was separated from the stria vascularis and spiral ganglion and split equally into the apex, middle, and base. The middle parts were removed and the apical and basal fragments were collected in separate tubes and trypsinized with prewarmed 0.125% trypsin/EDTA (Invitrogen) at 37°C for 8 min. Soybean trypsin inhibitor (10 mg/ml, Worthington Biochem) was then added to terminate the reaction in each apical and basal fragment. Cells were separated by mechanical trituration with blunt tips followed by pipetting up and down 80–100 times. Suspended cells were percolated through a 40 μm cell strainer (BD Biosciences). Dissociated cells from the apical and basal turns were sorted on a BD FACS Aria III using the GFP channel. Re-sort analysis, immunostaining, and qPCR were performed to evaluate the purity of the flow-sorted cells.

### Genotyping PCR and real-time qPCR

Transgenic mice were genotyped using genomic DNA from tail tips by adding 70 μl 50 mM NaOH, incubating at 98°C for 20–40min, and adding 7 μl 1M HCl. The genotyping primers were as follows: Lgr5: (F) CTG CTC TCT GCT CCC AGT CT; wild-type (R) ATA CCC CAT CCC TTT TGA GC; mutant (R) GAA CTT CAG GGT CAG CTT GC; tdTomato: wild-type (F) AAG GGA GCT GCA GTG GAG T; (R) CCG AAA ATC TGT GGG AAG TC; mutant (F) GGC ATT AAA GCA GCG TAT C; (R) CTG TTC CTG TAC GGC ATG G.

For quantitative polymerase chain reaction (qPCR), the Cells-to-cDNA II kit (Ambion; AM 1722) was used to extract total RNA and reverse transcribe it into cDNA using oligo(dT) primers. The FastStart Universal SYBR Green Master (ROX) kit (Roche) was used to perform qPCR on a BIO-RAD C1000 Touch thermal cycler. Each qPCR reaction was performed in triplicate with β-actin as the reference endogenous gene and analyzed using the ΔΔC_T_ method. Primer pairs were designed using the online Primer3 software. Lgr5 (F) CCT ACT CGA AGA CTT ACC CAG T; (R) GCA TTG GGG TGA ATG ATA GCA; Sox2 (F) GCG GAG TGG AAA CTT TTG TCC; (R) CGG GAA GCG TGT ACT TAT CCT T; Brn3.1 (F) CGA CGC CAC CTA CCA TAC C; (R) CCC TGA TGT ACC GCG TGA T; β-actin (F) GGC TGT ATT CCC CTC CAT CG; (R) CCA GTT GGT AAC AAT GCC ATG T.

### Culture of flow-sorted Lgr5+ cells from the apical and basal turns

Sorted apical Lgr5+ progenitors (ALPs) and basal Lgr5+ progenitors (BLPs) were cultured to a density of 50 cells/μl on laminin-coated plates using DMEM/F12 medium supplemented with N2 (1:100 dilution, Invitrogen), B27 (1:50 dilution, Invitrogen), heparin sulfate (50 ng/ml, Sigma), and the growth factors bFGF (10 ng/ml, Sigma), EGF (20 ng/ml, Sigma), and IGF-1 (50 ng/ml, Sigma) for 10 days. EdU (10 μM, Invitrogen) was included in the medium to measure cell proliferation ability.

### Sphere assay and differentiation assay

Sorted ALPs and BLPs were cultured separately at a density of 2 cells/μl in Costar ultra-low attachment dishes for 5 days using DMEM/F12 medium supplemented with N2, B27, heparin sulfate, and the growth factors bFGF, EGF, and IGF-1. Spheres were quantified before passaging to the next generation. For differentiation, neurospheres were plated on laminin-coated plates in DMEM/F12 medium for 5 days. EdU (10 μM) was used to label dividing cells. Differentiated neurospheres were fixed and analyzed by immunohistochemistry.

### Immunostaining and image acquisition

Cells and differentiated neurospheres were fixed in 4% PFA for 1 h at room temperature, followed by blocking with PBS (PH 7.4) containing 5% donkey serum, 0.1% Triton X100, 0.02% sodium azide (NaN3), and 1% bovine serum albumin (BSA) for 1 h at room temperature. This was followed by an overnight incubation with primary antibodies diluted in the same blocking solution at 4°C. Immunostaining was resumed by washing with PBS and incubating with secondary antibody (Invitrogen) for 1 h at room temperature. This was followed by washing and then mounting in antifade fluorescence mounting medium (DAKO). The antibodies used in this study were anti-Myosin7a (Proteus Bioscience) and anti-Sox2 (Santa Cruz Biotechnology). Cell proliferation was measured with the Click-it EdU imaging kit (Invitrogen). A Zeiss LSM 710 confocal microscope was used to capture images, and the images were analyzed using imageJ (NIH) and Photoshop CS4 (Adobe System).

### RNA extraction for GeneChip microarray

Approximately 5,000 Lgr5+ cells from the apical region and 5,000 Lgr5+ cells from the basal region were separately stored in RNALater for less than 48 hours at 4°C. Total RNA was extracted using the RNeasy micro kit (QIAGEN). The concentrations and purities of the RNA samples were analysed on a NanoDrop spectrophotometer (Isogen Life Science). Apical and basal RNAs were split into three fractions for separate replicates.

### GeneChip microarray

Gene expression profiles were obtained with the GeneChip Mouse Gene 2.0 ST Arrays with approximately 20–30 ng of total RNA obtained from ALPs and BLPs. Total RNA was amplified, labeled, and purified using the Encore biotin module of the NuGEN Ovation Pico WTA System V2 following the manufacturer's instructions to obtain biotin-labeled cDNA. Array hybridization and washes were performed using the GeneChip Hybridization, Wash and Stain Kit (Affymetrix) in a Hybridization Oven 645 (Affymetrix) and Fluidics Station 450 (Affymetrix). Slides were scanned by a GeneChip Scanner 3000 (Affymetrix) using the Command Console Software 3.1 (Affymetrix) with default settings. CEL files from the Mouse Gene 2.0 chips were normalized with the RMA algorithm in the Expression Console software.

### Data analysis

The whole-transcript arrays included probes to measure the expression of mRNA and long intergenic non-coding RNA transcripts. A total of 41,345 mouse RefSeq transcripts were included in the microarray according to information provided by the manufacturer. Means and standard deviations of normalized data were calculated. Paired *t*-tests were performed to compare average intensity values for each transcript from three repeated experiments. A value of *p* ≤ 0.05 was considered statistically significant. Annotation of the probe set was done using the file for the Mouse Gene 2.0 ST Array, Analyses. Because the expression of every transcriptional unit was measured by signal intensity, a cutoff level of 16.3 was chosen by averaging the signals of the antigenomic background probes from all six arrays.

## SUPPLEMENTARY FIGURE


