# Supplementary material for: Characterization of Lgr5+ progenitor cell transcriptomes in the apical and basal turns of the mouse cochlea
Source: Oncotarget. 2016 Apr 7;7(27):41123–41. doi: 10.18632/oncotarget.8636 (PMC5173047; doi:10.18632/oncotarget.8636)
Supplement: Supplementary file 1 [file oncotarget-07-41123-s001.pdf]

SUPPLEMENTARY FIGURE

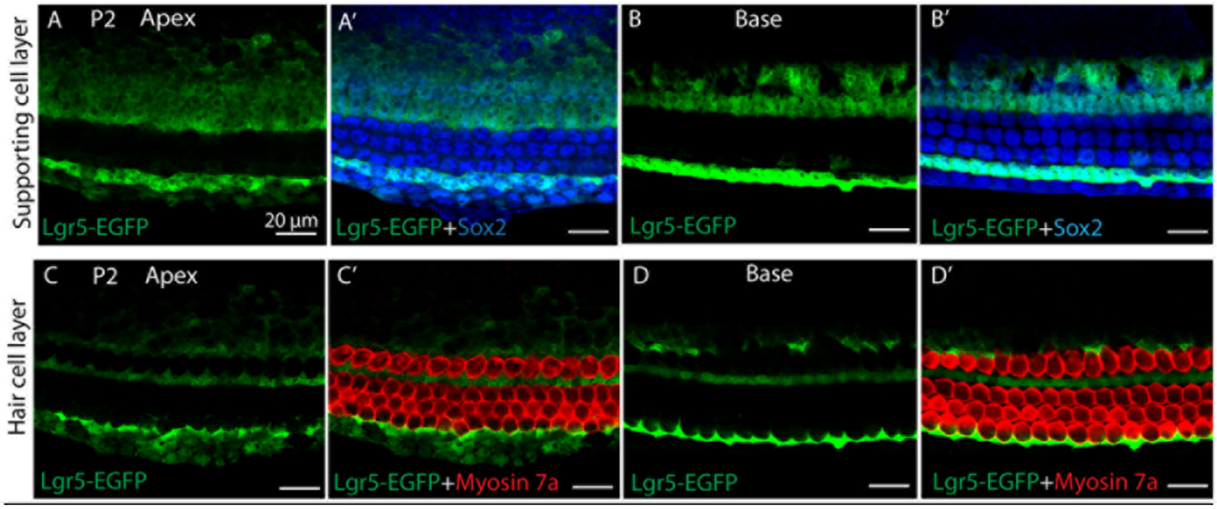

Supplementary Figure S1: The expression of Lgr5-EGFP in a subset of SCs in the apical and basal turns of the postnatal day 2 mouse cochlea. A-D. The Lgr5-EGFP+ SCs cells were observed in the third row of Deiters' cells, inner pillar cells, inner phalangeal cells, and the greater epithelium region (GER) in both the apical and the basal turn of the cochlea. However, compared to the basal turn, the apical region contained more Lgr5-EGFP+ cells in the GER. Lgr5-EGFP expression was not detected in the myosin 7a+ HCs. Scale bars are 20 µm.
